# Modulation of the Redox Potential and Electron/Proton Transfer Mechanisms in the Outer Membrane Cytochrome OmcF From *Geobacter sulfurreducens*

**DOI:** 10.3389/fmicb.2019.02941

**Published:** 2020-01-14

**Authors:** Liliana R. Teixeira, Cristina M. Cordas, Marta P. Fonseca, Norma E. C. Duke, Phani Raj Pokkuluri, Carlos A. Salgueiro

**Affiliations:** ^1^UCIBIO-Requimte, Departamento de Química, Faculdade de Ciências e Tecnologia, Universidade NOVA de Lisboa, Caparica, Portugal; ^2^LAQV, REQUIMTE, Departamento de Química, Faculdade de Ciências e Tecnologia, Universidade NOVA de Lisboa, Caparica, Portugal; ^3^Biosciences Division, Argonne National Laboratory, Lemont, IL, United States

**Keywords:** site-directed mutagenesis, electron transfer proteins, *c*-type cytochrome, redox-Bohr effect, cyclic voltammetry, nuclear magnetic resonance, X-ray crystallography

## Abstract

The monoheme outer membrane cytochrome F (OmcF) from *Geobacter sulfurreducens* plays an important role in Fe(III) reduction and electric current production. The electrochemical characterization of this cytochrome has shown that its redox potential is modulated by the solution pH (redox-Bohr effect) endowing the protein with the necessary properties to couple electron and proton transfer in the physiological range. The analysis of the OmcF structures in the reduced and oxidized states showed that with the exception of the side chain of histidine 47 (His^47^), all other residues with protonatable side chains are distant from the heme iron and, therefore, are unlikely to affect the redox potential of the protein. The protonatable site at the imidazole ring of His^47^ is in the close proximity to the heme and, therefore, this residue was suggested as the redox-Bohr center. In the present work, we tested this hypothesis by replacing the His^47^ with non-protonatable residues (isoleucine – OmcFH47I and phenylalanine – OmcFH47F). The structure of the mutant OmcFH47I was determined by X-ray crystallography to 1.13 Å resolution and showed only minimal changes at the site of the mutation. Both mutants were ^15^N-labeled and their overall folding was confirmed to be the same as the wild-type by NMR spectroscopy. The pH dependence of the redox potential of the mutants was measured by cyclic voltammetry. Compared to the wild-type protein, the magnitude of the redox-Bohr effect in the mutants was smaller, but not fully abolished, confirming the role of His^47^ on the pH modulation of OmcF’s redox potential. However, the pH effect on the heme substituents’ NMR chemical shifts suggested that the heme propionate P_13_ also contributes to the overall redox-Bohr effect in OmcF. In physiological terms, the contribution of two independent acid–base centers to the observed redox-Bohr effect confers OmcF a higher versatility to environmental changes by coupling electron/proton transfer within a wider pH range.

## Introduction

*c*-type cytochromes are key elements for the extracellular electron transfer processes in *Geobacter* species (Methé et al., 2003; Morgado et al., 2012). The genome of *Geobacter sulfurreducens* encodes for 128 *c*-type cytochromes out of which 31 are predicted to be located in the outer membrane (Methé et al., 2003). The outer membrane cytochrome F (OmcF) is one of these proteins. Genetic and proteomics studies have suggested that OmcF is an important protein for extracellular electron transfer in the respiratory pathways responsible for Fe(III) reduction and current production by *G. sulfurreducens* (Kim et al., 2005, 2008). However, the results obtained suggested that OmcF is not directly involved in these respiratory pathways being necessary for the transcription of the appropriate genes encoding for proteins directly involved in Fe(III) reduction (OmcB and OmcC) or electricity production in microbial fuel cells (OmcE and OmcS) (Kim et al., 2005, 2008).

OmcF is a 104 amino acids protein with a predicted small lipid anchor at the N-terminus formed by the first 19 residues, a soluble domain consisting of residues 20–104 and a low-spin *c*-type heme group with axial His-Met coordination (Pokkuluri et al., 2009). The reduced and oxidized structures of the soluble part of OmcF have been determined by NMR and X-ray crystallography (Pokkuluri et al., 2009; Dantas et al., 2017). The structure of OmcF showed high similarity to those of cytochromes *c*_6_ from photosynthetic algae and cyanobacteria, particularly from *Scenedesmus obliquus* (Schnackenberg et al., 1999) and *Monoraphidium braunii* (Banci et al., 1998). Although the amino acid sequence of OmcF shows a higher homology with the cytochrome *c*_6_ of *S. obliquus*, the geometry of the heme axial methionine is more similar to that of cytochrome *c*_6_ from *M. braunii* (Pokkuluri et al., 2009).

The oxidized and reduced structures of OmcF are globally similar (Dantas et al., 2017). However, local redox-linked conformational changes were identified, in particular for the polypeptide segments Ala^53^-Ile^62^, Asn^74^-Gly^78^, Glu^84^-Ala^90^, and the C-terminus region (residues Val^100^-Pro^104^). In addition, the analysis of the pH-dependence of the backbone and side chain NH NMR signals also identified important pH-linked conformational changes, particularly in the vicinity of the heme group (Dantas et al., 2017). The most affected NH signals near the heme were those from residues His^47^, Glu^49^, Leu^52^, and Gly^76^.

The redox potential of OmcF was determined by visible potentiometric redox titrations and cyclic voltammetry at pH 7 and 8 using the normal hydrogen electrode (NHE) as reference (Pokkuluri et al., 2009; Teixeira et al., 2018). The values obtained by the two techniques were similar: + 180 (pH 7) and + 140 mV (pH 8) by cyclic voltammetry and + 180 (pH 7) and + 127 (pH 8) by potentiometric redox titrations. The values obtained indicate that the redox potential of OmcF is significantly modulated in this pH range (pH 7–8). The observed pH modulation of the formal redox potential (known as the redox-Bohr effect) indicates that the protein is able to thermodynamically couple electron and proton transfer. A complete electrochemical study covering a wider pH range, between 3.3 and 9.0, was also carried out and showed that the redox potential only varies considerably in the physiological pH range for *G. sulfurreducens* cellular growth (between pH 6 and 8) (Teixeira et al., 2018). This study also permitted the determination of the *pK*_*a*_ values for the redox-Bohr center in the reduced (*pK*_*red*_ = 7.6) and oxidized (*pK*_*ox*_ = 6.7) states. However, the molecular determinant(s) responsible for the pH modulation of the OmcF redox potential have not yet been identified.

**FIGURE 1:**
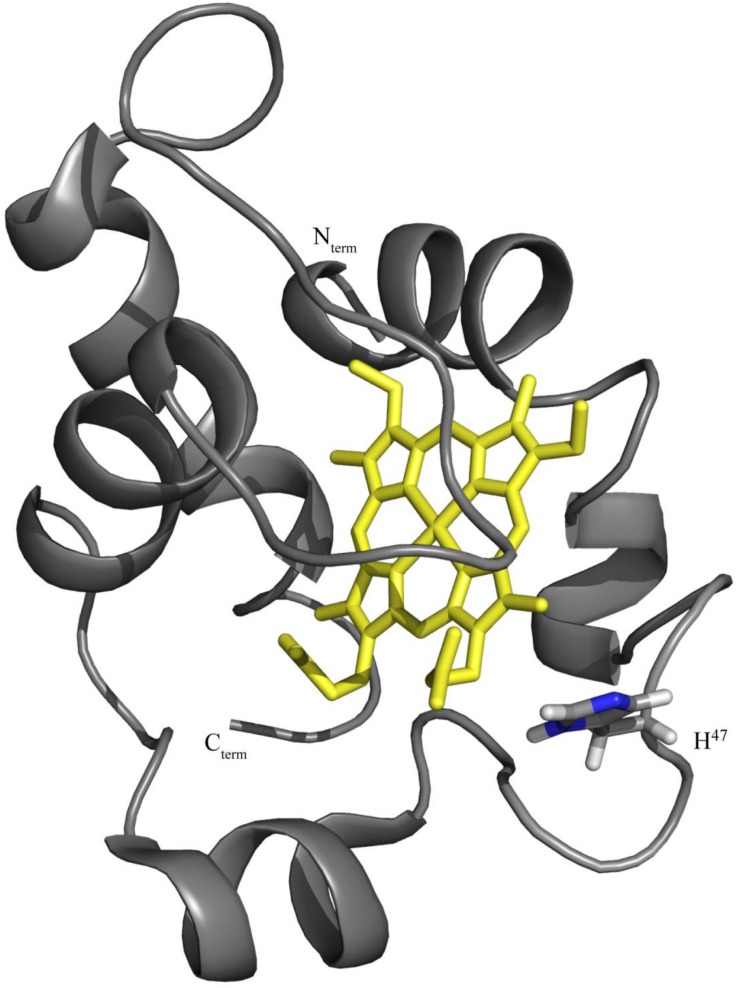
OmcF’s lowest energy solution structure [PDB code 5MCS (Dantas et al., 2017)] highlighting the location of residue His^47^. The OmcF polypeptide chain and the heme group are colored gray and yellow, respectively. The N- and C-termini, as well as side chain and H^α^ proton of His^47^ are labeled. Figure was generated with PyMOL (The PyMOL Molecular Graphics System, Version 1.3 Schrödinger, LLC).

The identification of the redox-Bohr center is important to elucidate the functional mechanism of OmcF and to contribute to the understanding of the extracellular electron transfer processes in *G. sulfurreducens*. The protonatable site of the imidazole ring of histidine 47 (His^47^) has been hypothesized as the redox-Bohr center based: (i) on the typical *pK*_*a*_ value for a histidine side chain and (ii) on its spatial location near the heme in the structure of OmcF (Figure 1; Dantas et al., 2017). To test this hypothesis, in the present work, we used site-directed mutagenesis to replace the His^47^ by isoleucine (OmcFH47I) and phenylalanine (OmcFH47F) residues. In both cases, the side chain of the substituting residues is not protonatable and, therefore, will permit the evaluation of the contribution of His^47^ to the observed redox-Bohr effect. Non-labeled (hereafter referred as natural abundance) and ^15^N-labeled mutants were produced. Their overall folding was confirmed to be the same as the wild-type cytochrome by NMR and their electrochemical characterization was carried out in the physiological pH range. In addition, the crystal structure of the OmcFH47I mutant was determined.

## Materials and Methods

### Site-Directed Mutagenesis

The residue His^47^ was replaced by isoleucine (OmcFH47I) and phenylalanine (OmcFH47F) using the NZYMutagenesis kit (NZYTech) and the pCK32 vector (encoding for the soluble part of OmcF formed by residues 20–104) as template (Pokkuluri et al., 2009). The necessary oligonucleotides were designed by the QuikChange Primer Design program (Agilent Technologies). For the OmcFH47I and OmcFH47F mutants, the following pairs of primers were used: 5′-agggtcttttccggg**atg**acggtgttgcctcc-3′/5′-ggaggcaacaccgt**cat**cccggaaaagaccct-3′ (OmcFH47I) and 5′-agggtc ttttccggg**aag**acggtgttgcctcc-3′/5′-ggaggcaacaccgt**ctt**cccggaaaagacc ct-3′ (OmcFH47F). All primers were synthesized by Invitrogen and the mutations were confirmed by DNA sequencing (STAB VIDA).

### Expression and Purification of OmcF and Mutants

Natural abundance and ^15^N-labeled proteins were expressed and purified as previously described (Fernandes et al., 2008; Pokkuluri et al., 2009). A summary of the procedure is presented here. *Escherichia coli* cells BL21 (DE3) containing the plasmids pEC86 (encoding for the expression of *c*-type cytochrome maturation proteins) (Arslan et al., 1998) and pCK32 were grown in 2xYT medium to an OD_600 nm_ value between 1.5 and 1.8. From this point forward, distinct protocols were used for production of natural abundance and labeled proteins. In the first case, the expression was induced with 20 μM isopropyl β-D-thiolgalactopyranoside (IPTG), and the cultures were incubated overnight at 30°C. In the second case, to produce ^15^N-labeled proteins, cells were collected by centrifugation and transferred to M9 minimal medium supplemented with ^15^NH_4_Cl and α-aminolevulinic acid. Protein expression was then induced with 100 μM IPTG and the cultures were incubated overnight at 30°C.

In both cases, cells were first harvested by centrifugation (6400 ×*g* for 20 min) and lyzed using a buffer containing 100 mM Tris-HCl (pH 8), 0.5 mM EDTA, 20% sucrose, and 0.5 mg/mL of lysozyme. The periplasmic fraction was then recovered by centrifugation (14,700 ×*g* for 20 min) followed by an ultracentrifugation step (225,000 × for 1 h). This fraction was dialyzed against 20 mM sodium acetate (pH 5). After the dialysis step, the protein was loaded onto cation-exchange columns (2 × 5 mL Econo-Pac High S cartidges, Bio-Rad) pre-equilibrated with the same buffer and eluted with a linear sodium chloride gradient (0–200 mM). The fractions containing the targeted protein were then loaded onto a XK 16/70 Superdex^TM^ 75 prep grade column (GE Healthcare Life Sciences) pre-equilibrated with the same buffer. The protein’s purity was accessed by dodecyl sulfate polyacrylamide gel electrophoresis (SDS-PAGE) stained with BlueSafe (NZYTech) and its concentration determined by visible spectroscopy using an absorption molar coefficient of 23.8 × 10^3^ M^–1^cm^–1^ (Lukat et al., 2008).

### NMR Studies

NMR spectra were acquired on a Bruker Avance 600 MHz spectrometer with a triple-resonance cryoprobe at 25°C. To assist the assignment of the backbone and side chain NH signals in each mutant, ^15^N-labeled samples were prepared in 45 mM sodium phosphate (pH 7) with 100 mM final ionic strength in 92% H_2_O/8% ^2^H_2_O. Natural abundance samples of the mutants were prepared in the same buffer to assist the assignment of the heme substituent signals. OmcF wild-type samples were also prepared in the same buffer (pH 6.1 and 9.4) in ^2^H_2_O (99.9%) to study the pH dependence of the heme substituents’ signals.

Reduction of the proteins was achieved by adding an equimolar solution of sodium dithionite, after degassing the samples with a continuous flow of argon. The full reduction of the samples was confirmed by 1D ^1^H NMR. 2D ^1^H,^15^N-HSQC spectra were acquired for ^15^N-labeled samples, whereas 2D ^1^H, ^1^H-TOCSY (60 ms) and 2D ^1^H, ^1^H-NOESY (80 ms) were acquired for natural abundance samples.

The water signal was used to calibrate the ^1^H chemical shifts. ^15^N chemical shifts were calibrated using indirect referencing (Wishart et al., 1995). The data were processed using TOPSPIN (Bruker Biospin, Karlsruhe, Germany) and analyzed with Sparky (TD Goddard and DG Kneller, Sparky 3, University of California, San Francisco, CA, United States).

### Electrochemical Studies

The cyclic voltammetry measurements were performed inside a Faraday cage using a three electrodes’ configuration in a single compartment electrochemical cell, as described previously (Teixeira et al., 2018). Briefly, a neomycin sulfate (3 μL of a 2 mM stock) and protein solutions (5 μL of a 255 μM stock) were placed on the working electrode, followed by evaporation at room temperature (solvent casting technique). The thin layer procedure was implemented by the entrapment of the protein using a cellulose membrane (spectra/Pro) with a 6 to 8 kDa cut-off. The electrodes were immersed into a buffer solution of 32 mM sodium phosphate (pH 6, 7, and 8) with 100 mM final ionic strength, previously degassed with a continuous flow of argon. All assays were carried out at room temperature and at least three independent replicates were performed for each assay at scan rates between 2.5 and 100 mVs^–1^. From the midpoint redox potential obtained for each scan rate using the second cycle [using *E^0^’* = (*E_*pa*_* + *E*_*pc*_)/2], the average and standard deviation values were calculated. Controls were performed applying the same methodology but in the absence of protein. The measured redox potential values were corrected for the NHE (Sawyer et al., 1995; Liu et al., 1997; Lindgren et al., 2000).

#### Crystallization, Data Collection, and Structure Determination

Crystallization trials for both mutants were carried out by hanging drop vapor diffusion method at room temperature using protein sample concentrations of 20 mg/mL. Attempts to grow crystals using the same conditions as the wild-type OmcF (1.2 M trisodium citrate dihydrate, 0.1 M Tris pH 8.5) were unsuccessful. However, very thin needle clusters in case of OmcFH47I were obtained using the conditions reported for OmcF consisting of an N-terminus Strep-tag II (Lukat et al., 2008). The drops consisted of 1 μL of protein plus 1 μL precipitating reagent [1.8 M ammonium sulfate, 0.1 M HEPES pH 7.5, and 2%(v/v) PEG400] equilibrated over 0.5 mL of reagent solution in the well. The crystals were optimized for data collection by microseeding technique. A thin needle cluster grown from the above-mentioned condition was placed on a glass slide in a 10 μL drop of reservoir solution. Viewing under an optical microscope, this crystal cluster was crushed with a razor blade. The droplet containing the pieces of the crushed crystals was collected from the glass slide. This solution is named seed I. The glass slide was then washed with another fresh 10 μL drop of well solution and collected (named seed II solution). New crystallization trials were conducted using the same conditions as above, except hanging drops now consisted of 1 μL of protein, 1 μL well reagent, and 0.2 μL seed solution. Best crystals for OmcFH47I were obtained from a drop supplemented with seed II solution. Microseeding also yielded crystals as needle clusters. A piece cleaved from a cluster was used for data collection. In case of OmcFH47F mutant, seeding the crystallization drops with OmcFH47I seed solution was unsuccessful.

The X-ray diffraction data were collected at the 22ID beam line of the SER-CAT, Advanced Photon Source (Argonne, IL, United States). X-ray diffraction data were collected to a high resolution of 1.13 Å. Data reduction and scaling was achieved with the program HKL 2000 (Otwinowski and Minor, 1997). Structure solution was achieved by molecular replacement using the wild-type OmcF coordinates [PDB code 3CU4 (Pokkuluri et al., 2009)], after removing the side chain atoms beyond C_β_ of His^47^ using the Phaser-MR (McCoy et al., 2007) routine within Phenix package (Adams et al., 2010). Auto-build routine within Phenix was used for model building. The program Coot (Emsley and Cowtan, 2004) was used to inspect the electron density maps and make the necessary adjustments in the model and solvent molecules. Refinement was carried out by the program Phenix. The final model included residues 23–104, one heme-*c*, 91 water molecules, and a sulfate ion. The data collection and refinement statistics are presented in Table 1.

**TABLE 1:** Crystallographic parameters and refinement statistics for OmcFH47I mutant.

**Crystal and data parameters**	
Unit cell dimensions	*a* = 38.186 Å *b* = 39.019 Å, *c* = 49.218 Å
Space group	P2_1_2_1_2_1_
#mol/AU	1
V_M_ (Å^3^/Da) (% solvent)	2.1 (43)
Wavelength (Å)	0.91840
Resolution^a^ (Å)	50-1.13 (1.15-1.13)
R-merge^a^	0.054 (0.304)
CC_1/2_^a^	0.99 (0.96)
Redundancy^a^	11 (6)
Completeness^a^ (%)	99 (95)
Mean I/σ(I)^a^	42 (5)
**Refinement**	
Program used	Phenix
Resolution range (Å)	23.9-1.13
Number of reflections	18212
R-factor	0.126
R-free^b^	0.153
**Number of non-hydrogen atoms (mean *B*-factor, Å^2^)**	
Protein	636 (12.9)
Heme	43 (10.1)
Solvent	96 (24.6)
**Rmsd**	
Bonds (Å)	0.013
Bond angles (°)	1.2
**Ramachandran plot (%)**	
Favored	99
Allowed	1
PDB code	6U97

*^*a*^The values in parentheses correspond to the highest resolution shell. ^*b*^R-free calculated with 5% of the reflections.*

## Results and Discussion

### Impact of the Mutations on the Global Folding of the Protein

The protein yields obtained for each mutant (OmcFH47I and OmcFH47F) were similar to the ones obtained for the wild-type protein, approximately 10 and 3 mg of protein per liter of cell culture for natural abundance and ^15^N-labeled proteins, respectively. The UV–visible spectra of the mutants showed similar features compared to the wild-type protein (Supplementary Table S1).

**FIGURE 2:**
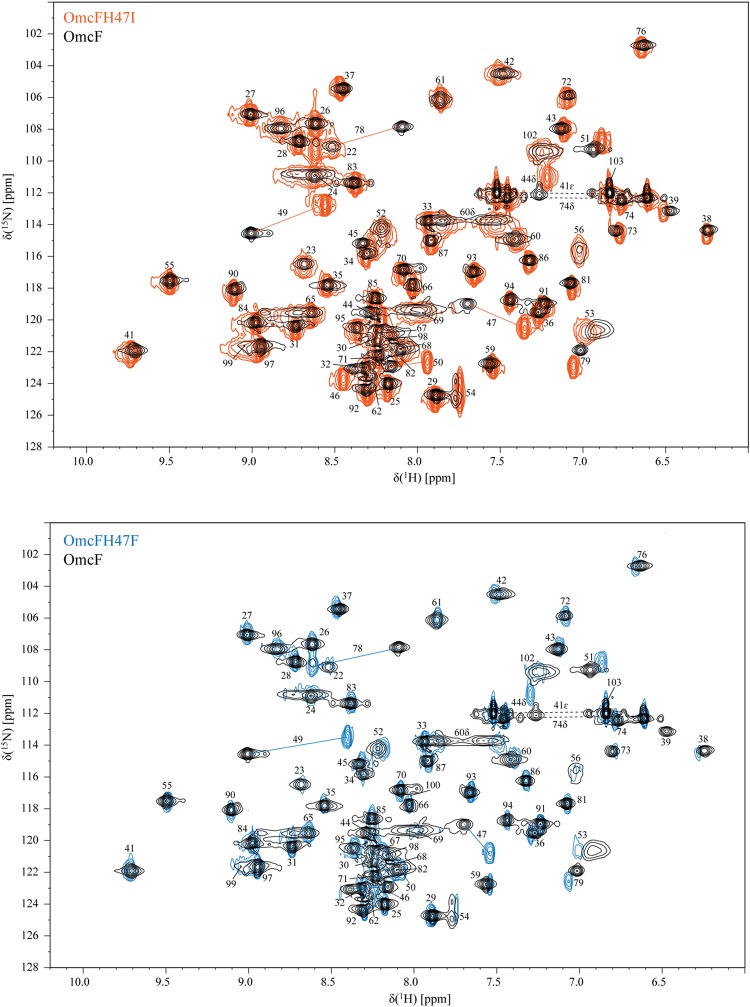
2D ^1^H,^15^N-HSQC NMR spectra of OmcF (black contours), OmcFH47I (orange contours), and OmcFH47F (blue contours) in the reduced state (25°C, pH 7). The most affected signals in the mutants, compared to the wild-type spectrum, are connected by a straight line. Dashed lines correspond to side chains of amino acids.

**FIGURE 3:**
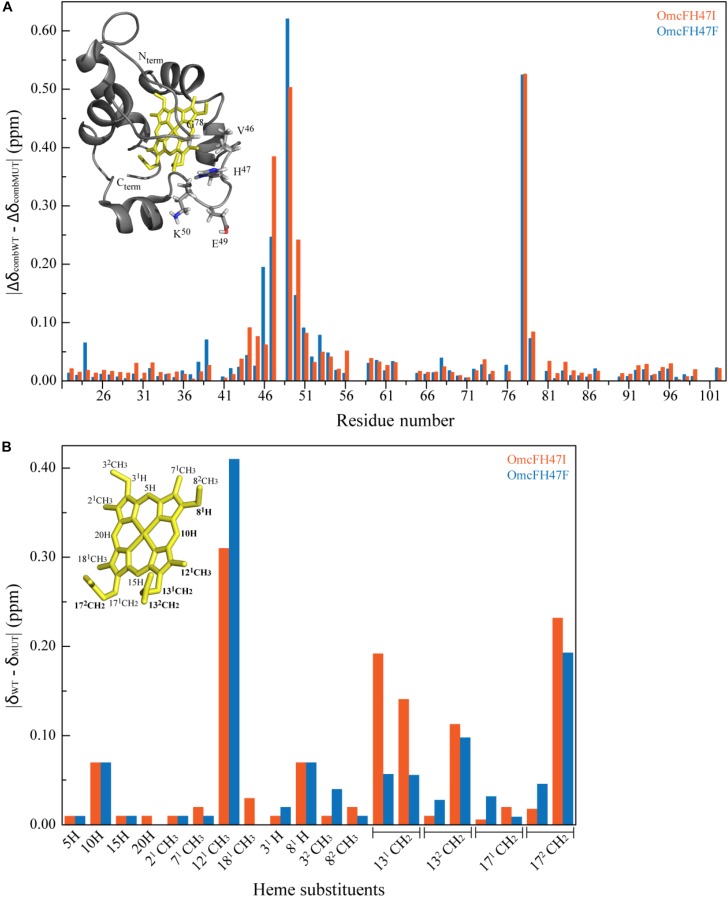
Effects on the polypeptide and heme substituent’s NMR signals caused by the replacement of His^47^ by isoleucine or phenylalanine. **(A)** Comparison between the combined ^1^H and ^15^N chemical shifts observed in the 2D ^1^H,^15^N HSQC NMR spectra of OmcF mutants (Δδ_comb_MUT) and those of OmcF (Δδ_comb_WT). The differences were calculated using the equation Δδ_comb_ = [(Δδ_H_)^2^ + w_i_(Δδ_N_)^2^]^1/2^, where Δδ_H_ is the difference between ^1^H shifts, Δδ_N_ the difference between ^15^N shifts, and w_i_ = ∣γ^15^_N_∣/∣γ^1^_H_∣ a weighting factor that accounts for the differences in nuclei sensitivity (Schumann et al., 2007). The color code of the NMR spectra indicated in Figure 2 was used for each mutant: OmcFH47I (orange bars) and OmcFH47F (blue bars). The most affected residues are shown in the solution structure of cytochrome OmcF in the inset. **(B)** Comparison of the heme ^1^H chemical shifts observed in the 2D ^1^H NOESY NMR spectra of OmcF mutants (δ_MUT_) and those of wild-type OmcF (δ_WT_). The heme substituents are numbered according to the IUPAC-IUB nomenclature (Moss, 1988) and the most affected heme substituent signals are highlighted in bold in the heme diagram. In both panels the structural data correspond to OmcF’s lowest energy solution structure [PDB code 5MCS (Dantas et al., 2017)] generated with PyMOL (The PyMOL Molecular Graphics System, Version 1.3 Schrödinger, LLC).

The NMR chemical shifts of the nuclei of the molecule are very sensitive to changes in their chemical environment and, consequently, can be explored to fingerprint the overall structure of the proteins in solution. Thus, before undertaking the electrochemical characterization of the mutants, the impact of each mutation on the protein conformation was evaluated by 2D ^1^H,^15^N-HSQC NMR experiments. The backbone and side chain NH signals of each mutant were assigned (Figure 2) using the same methodology described for the wild-type protein (Dantas et al., 2015). The comparison of the spectra obtained for the wild-type and the mutants showed a similar dispersion of signals, indicating that the overall folding is maintained (Figure 2). The analysis of the ^1^H and ^15^N combined chemical shift variation showed that the residues located in the polypeptide segment Val^46^-Lys^50^ and Gly^78^ were the most affected (Figure 3A). The polypeptide segment Val^46^-Lys^50^ includes the mutated residue (Ile^47^ and Phe^47^ for OmcFH47I and OmcFH47, respectively) and a proline residue (Pro^48^). This residue does not possess NH due to its cyclic nature and for this reason no chemical shift variation was observed. On the other hand, Gly^78^ is closely located to Val^46^, whose side chain, together with the imidazole ring of His^47^, forms a pocket around the heme methyl 12^1^CH_3_ (see inset in Figure 3A). Thus, the replacement of His^47^ residue introduced only small rearrangements at the vicinity of the mutation site, without affecting the global folding of the protein. Both mutants showed a similar variation of their chemical shift suggesting that the local rearrangements are comparable. Following the analysis of the impact of the mutated residues in the backbone and side chain NH signals, their impact was further evaluated regarding the heme ^1^H NMR signals. The heme signals were assigned using 2D ^1^H, ^1^H-TOCSY and 2D ^1^H, ^1^H-NOESY NMR spectra, as described for the wild-type protein (Dantas et al., 2015). The assignment of these signals and their comparison with those obtained for the wild-type protein are indicated in Supplementary Table S2 and Figure 3B, respectively. In both mutants, the most affected signal is the heme methyl 12^1^CH_3_, which is the closest to the mutated residue (*cf*. insets in Figures 3A,B). The other affected heme signals, although to a smaller extent, correspond to propionate protons (13CH_2_ groups), the meso proton 10H and the thioether proton 8^1^H, which are allocated to the heme face containing the methyl 12^1^CH_3_ (see inset in Figure 3B). Therefore, the heme proton signals showing the largest chemical shift variations are in the vicinity of most affected residues, namely, the polypeptide segment Val^46^-Lys^50^ and Gly^78^ (*cf*. insets in Figures 3A,B), which further confirmed that the small conformational changes caused by the amino acid replacements are restricted to the neighboring regions of the mutated residue.

### Effect of the Mutations on the Redox-Bohr Center Properties

The redox potential values of cytochromes can be modulated by the solution pH which would be functionally relevant within a physiological pH range. This modulation is designated redox-Bohr effect, in analogy with the Bohr effect in the hemoglobin (Perutz, 1989). From a simple electrostatic view of the heme iron charge, the reduced form is expected to be stabilized by the protonation of the redox-Bohr center while its deprotonation stabilizes the oxidized form.

**FIGURE 4:**
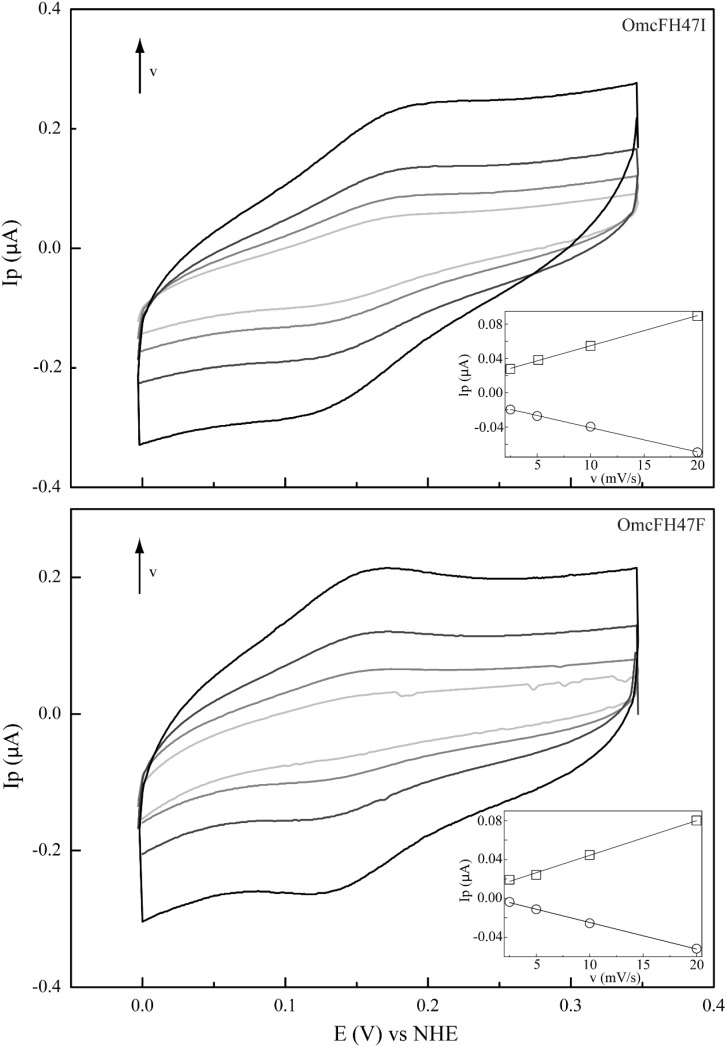
Cyclic voltammograms of mutants OmcFH47I **(top)** and OmcFH47F **(bottom)** at scan rates (ν) from 2.5 to 20 mV s^–1^ (pH 7). Inset: peak current as a function of the scan rate. Anodic and cathodic peak currents are represented by squares and circles, respectively.

Previous studies have shown that the redox potential of OmcF is strongly modulated in the pH range 6–8, i.e., at the physiological range for the *G. sulfurreducens* growth (Pokkuluri et al., 2009; Teixeira et al., 2018). Thus, in the present work, after confirming that only local conformational changes were observed in the region of the mutated residues, the redox potential values of the OmcF mutants were measured by cyclic voltammetry in the same pH range. For proper comparison, measurements for the wild-type protein were carried out in the same experimental conditions as the mutants in the present study and are reported in Table 2. The cyclic voltammograms were recorded in the potential window between 0 and 0.35 V, with scan rates between 2.5 and 100 mVs^–1^. At all pH values, well-defined redox pairs were visible and, by comparison with the controls, were assigned to the OmcF heme group. The data obtained for the OmcF mutants at pH 7 are illustrated in Figure 4 (cyclic voltammograms obtained for OmcF at pH 7 and corresponding control are shown in Supplementary Figure S1). The signals show a quasi-reversible electrochemical behavior between the scan rates 2.5–20 mVs^–1^, just like the wild-type cytochrome (Teixeira et al., 2018). The quasi-reversible electrochemical behavior was verified through the usual criteria for the thin layer regime, namely: the ratio of the cathodic (*I*_*pc*_) and anodic (*I*_*pa*_) peak current intensities are approximately 1; the *I*_*pc*_ and *I*_*pa*_ are linearly proportional to the scan rate, and the separation between the cathodic (*E*_*pc*_) and anodic (*E*_*pa*_) peak potential (Δ*Ep*) increases with the applied scan rate. The estimated thin layer thickness was in average 2.5 μm (Brett and Brett, 1993; Bard and Faulkner, 2001; Correia dos Santos et al., 2003; Santos et al., 2015). The formal potential values of OmcFH47I and OmcFH47F mutants were determined by the average of the anodic and cathodic peak potentials (at the point of maximum current intensity) and are indicated in Table 2. As it is possible to observe, the formal potential values for both mutants were lower than the value found for the wild-type cytochrome. This indicates that the oxidized form in the mutants is more stabilized as expected by the replacement of the positively charged side chain of His^47^ in the wild-type by an uncharged side chain in both mutants.

**TABLE 2:** Formal potential values (*versus* NHE) for OmcF and His^47^ mutants determined by cyclic voltammetry at pH 6, 7, and 8.

	**E^0’^ (mV)**		
	**pH 6**	**pH 7**	**pH 8**
OmcFH47I	179.8 ± 2.6	153.0 ± 1.8	125.0 ± 4.5
OmcFH47F	178.0 ± 1.6	146 ± 3.0	120.3 ± 3.5
OmcF	214.5 ± 3.9	179.3 ± 2.0	136.3 ± 2.2

*The respective standard errors are indicated.*

As observed for the wild-type cytochrome, the redox potential values of the mutants were also pH-dependent. However, the difference between the redox potential values of the mutants and those of the wild-type decreases with the increase in pH, which indicates that the replacements have a smaller impact on the redox properties of the heme group at higher pH. Overall, the data obtained suggested that the redox-Bohr center is protonated at pH 6 and deprotonated at pH 8. This is also in agreement with the *pK*_*a*_ values previously determined for the redox-Bohr center (*pK*_*ox*_ = 6.7; *pK*_*red*_ = 7.6) (Teixeira et al., 2018) and explains the higher impact of the substitutions at pH 6. The additional positive charge in the redox-Bohr center at pH 6 stabilizes the reduced state (higher redox potential values) whereas the loss of the positive charge at higher pH facilitates the oxidation of the heme and stabilizes the oxidized state (lower redox potential values). This is also reflected in the differences observed for the redox potential values of the mutants compared to the wild-type cytochrome at pH 6 (34.7 and 36.5 mV for OmcFH47I and OmcFH47F, respectively) and 8 (11.3 and 16.0 mV for OmcFH47I and OmcFH47F, respectively). In fact, in the wild-type at pH 6, the side chain of His^47^ is protonated and positively charged, whereas at pH 8, the side chain of His^47^ is deprotonated and uncharged. In the mutants, the replacements do not introduce any charge variation in the vicinity of the heme group at both pH values.

From the analysis of Table 2, it is also clear that the pH dependence of the redox potential values is smaller in the mutants compared to the wild-type cytochrome but confirms unequivocally the involvement of His^47^ in the pH modulation of the redox potential of OmcF. However, the fact that the redox-Bohr effect was not completely abolished by the replacement of His^47^ by non-protonatable residues, suggested that additional acid group(s) might be involved in the global redox-Bohr effect.

### Structural Probe of the Redox-Bohr Center in OmcF

The analysis of the OmcF structures showed that, in addition to the side chain of His^47^, other protonatable groups include the N- and C-termini, six arginine, two aspartic acid, five glutamic acid, one histidine, two lysine, two tyrosine residues, and the heme propionate groups at positions 13 (P_13_) and 17 (P_17_). With the exception of heme propionate groups, all other protonatable groups are distant from the heme iron, and therefore are unlikely to affect the redox potential of OmcF. Since the heme propionates are the best additional candidates for the redox-Bohr effect, we evaluated the pH dependence of the heme substituent signals of OmcF in the reduced state. The assignment of the entire set of the heme substituents in the oxidized state is more complex compared to the reduced state due to the paramagnetic effect of the heme unpaired electron (Salgueiro et al., 1997). This often impairs the full assignment of the heme signals in the oxidized state, which is in fact the case for OmcF. Because the assignment of the heme substituent signals of OmcF was fully obtained for the reduced state at pH 7 (Dantas et al., 2015), this state was selected to evaluate the pH dependence of the heme substituent NMR signals. Considering the *pK*_*a*_ value of the redox-Bohr center in the reduced state (*pK*_*red*_ = 7.6) (Teixeira et al., 2018), the pH values of 6.1 and 9.4 were selected to ensure a proper comparison of the heme substituent chemical shifts when the redox-Bohr center is protonated and deprotonated, respectively.

**FIGURE 5:**
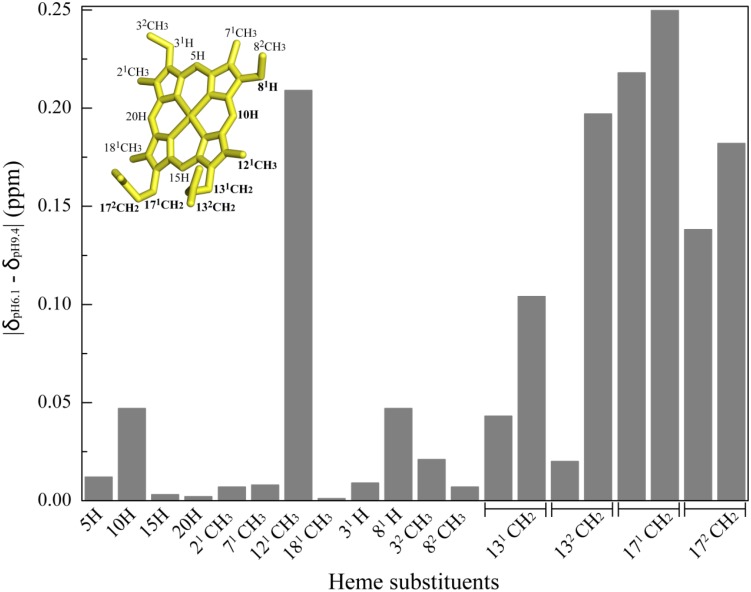
Variation of OmcF’s heme substituent’s proton chemical shifts at pH 6.1 (δ_pH__6__.__1_) and 9.4 (δ_pH__9__.__4_). The heme substituents are numbered according to the IUPAC-IUB nomenclature (Moss, 1988) and the most affected heme substituent signals are highlighted in bold in the heme diagram. The heme group in the inset was taken from OmcF’s lowest energy solution structure [PDB code 5MCS (Dantas et al., 2017)] and was generated with PyMOL (The PyMOL Molecular Graphics System, Version 1.3 Schrödinger, LLC).

The assignment of the heme substituent signals at pH 6.1 and 9.4 was carried out using the same strategy as previously described at pH 7 (Dantas et al., 2015) and are listed in Supplementary Table S3. The variation of the heme substituent’s chemical shifts at pH 6.1 and 9.4 is indicated in Figure 5 and shows that the heme propionate groups and methyl 12^1^CH_3_ are the most affected ones. However, the pH dependence of the heme propionates is quite distinct. Indeed, while the four protons of P_17_ were clearly affected by the pH, only one proton from P_13_ heme was affected. Such behavior, together with the fact that the heme methyl 18^1^CH_3_ is essentially pH independent, suggests that P_13_ is the best additional candidate for contribution to the redox-Bohr effect. In summary, electrochemical studies were carried out on OmcFH47I and OmcFH47F mutants and the pH dependence of the heme substituent NMR signals indicated that the two main contributors for the redox-Bohr effect in OmcF are the side chain of His^47^ and the heme propionate group P_13_. The deprotonation/protonation of the histidine side chain explains the large chemical shift variation on heme methyl 12^1^CH_3_ that extends also to heme protons 10H and 8^1^H, while the deprotonation/protonation of P_13_ is responsible for the significant variations observed for the chemical shift of the P_17_ protons (Figure 5).

### Crystal Structure of OmcFH47I

The OmcFH47I mutant crystallized in the same space group as the wild-type OmcF but with a slight difference in the unit cell dimensions resulting in a 1% decrease in unit cell volume of the mutant crystals. The overall structure of the OmcFH47I mutant in the oxidized state is very close to that of the wild-type with an overall root-mean-square deviation (rmsd) of 0.4 Å for all C_α_ atoms (residues 23–104). A C_α_ carbon trace of the overlap of the OmcFH47I mutant structure on the wild-type OmcF is shown in Supplementary Figure S2. The electron density for the side chain of Ile^47^ clearly showed two conformations, refined at occupancies of 0.7 and 0.3. The two conformations of the isoleucine side chain differed primarily in the location of the CD1 methyl group. Deviations higher than the overall rmsd (ranging from 0.6 to 0.8 Å) were observed near the mutation site from residues 47–50, and from residues 76 to 78, which are in line with the analysis of the ^1^H and ^15^N combined NMR chemical shift variation (Figure 3A). Deviations ranging from 1.0 to 1.3 Å were also seen in residues 84–86, which are distant from the heme, caused by different interactions across the crystal interface.

**FIGURE 6:**
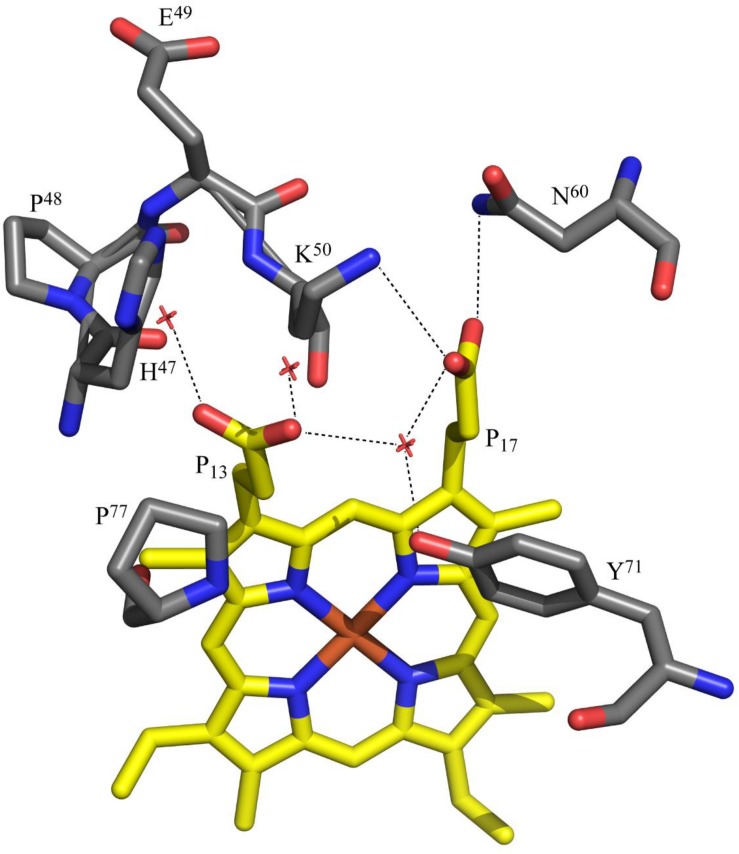
Polar interactions involving the heme propionates of OmcF. The propionates are labeled according to IUPAC nomenclature. The structural data corresponds to OmcF native structure [PDB code 3CU4 (Pokkuluri et al., 2009)] and was generated with PyMOL (The PyMOL Molecular Graphics System, Version 1.3 Schrödinger, LLC).

The replacement of the polar histidine residue by non-polar isoleucine did not cause any significant changes in the interactions of the heme with the protein. The isoleucine side chain(s) forms van der Waals contact(s) with the heme atoms. The propionate D (P_13_), which is closest to the mutation site, forms water mediated hydrogen bonds with Tyr^71^ and the other propionate in both wild-type and mutant structures. It also hydrogen bonds with other water molecules. Although this propionate can hydrogen bond with the side chain of His^47^ in the wild-type OmcF structure, this was not observed. On the other hand, the propionate A (P_17_) forms a salt bridge with Lys^50^ (NZ) with one of its carboxyl oxygen atoms and hydrogen bonds with Asn^60^ (ND2) with the other oxygen atom. These interactions involving the P_17_ carboxyl oxygen atoms are observed in both native and OmcFH47I mutant structures. The interactions formed by the heme propionates are shown in Figure 6.

#### Heme Propionate P_13_ and His^47^ Are the Redox-Bohr Centers in OmcF

The observed hydrogen bond network established by the heme propionate groups further supports the conclusion that P_13_ contributes to the global redox-Bohr effect in OmcF. In fact, it is common that the heme propionic acids ionize with *pK*_*a*_ values in the region 5–6 (Moore et al., 1980), and have been suggested as the groups responsible for the redox-Bohr effect in several monoheme cytochromes (Moore et al., 1980; Leitch et al., 1984; Costa et al., 1992; Santos et al., 2015). In the oxidized crystal structures of wild-type OmcF [PDB code, 3CU4 (Pokkuluri et al., 2009)] and in the OmcFH47I mutant (present work), one of the carboxyl atoms of the heme propionate P_17_ forms a salt bridge with NZ of Lys^50^ and the other carboxyl oxygen atom hydrogen bonds with ND2 of Asn^60^. Thus, this propionate is poised to be a hydrogen bond acceptor with both its carboxyl oxygen atoms, which stabilizes its deprotonated form. The stabilization offered by salt bridge with Lys^50^ and another hydrogen bond with Asn^60^ promotes to keep the *pK*_*a*_ of this propionate group (P_17_) lower than the other propionate group (P_13_). The propionate P_13_ on the other hand does not form any interactions with the protein in the crystal structures. It is somewhat more exposed to solvent and only interacts with water molecules located in the crystal. This propionate is close to the side chain of His^47^ in the wild-type OmcF structure but does not form a hydrogen bond with it. In the solution structure of OmcF [PDB code, 5MCS (Dantas et al., 2017)], the carboxyl group of propionate P_13_ is positioned between the side chain of His^47^ and the main chain oxygen atom of Pro^77^. In majority of the conformations, the propionate P_13_ forms two hydrogen bonds via its two carboxyl oxygen atoms, one with ND1 atom of His^47^ and another with the main chain oxygen atom of Pro^77^. The latter interaction will be unfavorable if this propionate is not in the protonated (neutral) form. The interaction between propionate P_13_ and the main chain oxygen of Pro^77^ is not seen in the crystal structure due to the intermolecular interactions with a neighboring molecule in the crystal. Alternatively, the difference could be related to redox conformational changes between the two structures, namely, crystal (oxidized) and solution (reduced). Therefore, we propose that the propionate P_13_ has a *pK*_*a*_ in the physiological range and, together with His^47^, the two acid–base groups contribute to extend the range of the overall redox-Bohr effect in OmcF. Upon removal of His^47^ in the OmcFH47 mutants, the magnitude of the redox-Bohr effect observed is approximately 30% less (54.8 and 57.7 mV for OmcFH47I and OmcFH47F, respectively, compared to 78.2 mV in OmcF – see Table 2).

## Conclusion

The cytochrome OmcF from the bacterium *G. sulfurreducens* showed an important pH modulation of the heme reduction potential (redox-Bohr effect) in physiological range for *G. sulfurreducens* cellular growth. The spatial localization of His^47^ and the properties of its side chain suggested it as a good candidate for the redox-Bohr center in OmcF. This hypothesis was addressed in the present work by replacing the His^47^ with the non-protonatable residues isoleucine and phenylalanine. The global folding of the mutants was assessed by NMR spectroscopy and the comparison of the polypeptide and heme NMR signals showed that both the mutants were properly folded and that only local conformational changes were observed in the vicinity of the mutated residue regions. The crystal structure of OmcFH47I mutant determined at a high resolution also showed that the mutation did not affect the structure. Electrochemical cyclic voltammetry studies carried out for both the mutants, within the physiological pH range, showed that the redox potential values and the redox-Bohr effect were smaller compared to the wild-type cytochrome. This unequivocally confirms the role of His^47^ in the pH modulation of the OmcF heme redox potential and electron/proton transfer mechanisms. However, the redox-Bohr effect was not fully abolished in the mutants (approximately 30% less in the mutants) suggesting the existence of another redox-Bohr center in OmcF, which was attributed to the heme propionate P_13_. Therefore, these two acid–base groups with *pK*_*a*_ in the physiological range (heme propionate P_13_ and His^47^) contribute to the overall observed redox-Bohr effect. Given the cellular location of OmcF at the outer membrane, the existence of two independent acid-base centers that contribute to redox-Bohr effect may permit the protein to be functionally active in a wider pH range, in response to environment changes. This study sheds light on how the cytochromes can extend the modulation of the redox potential within the physiological pH range not only through the interactions of their heme propionate groups with the neighboring protein atoms but also by utilizing strategic placement of additional protonatable residues near the heme. Delineation of such an intricate heme–protein interaction network is vital to a clearer understanding of the extracellular electron transfer processes mediated by these bacteria and the central role played by the multitude of cytochromes encoded within their genomes.

## Data Availability Statement

The datasets generated for this study can be found in the PDB code 6U97.

## Author Contributions

CS and PP conceived, designed, and supervised the project. LT and MF acquired and analyzed the NMR data. LT and CC acquired and analyzed the electrochemical data. ND and PP acquired and analyzed the X-ray data. LT, CC, PP, and CS wrote the manuscript.

## Conflict of Interest

The authors declare that the research was conducted in the absence of any commercial or financial relationships that could be construed as a potential conflict of interest.
