# Supplementary material for: Modulation of the Redox Potential and Electron/Proton Transfer Mechanisms in the Outer Membrane Cytochrome OmcF From Geobacter sulfurreducens
Source: Front Microbiol. 2020 Jan 14;10:2941. doi: 10.3389/fmicb.2019.02941 (PMC6971198; doi:10.3389/fmicb.2019.02941)

**Modulation of the redox potential and electron/proton transfer mechanisms in the outer membrane cytochrome OmcF from *Geobacter sulfurreducens***

Liliana R. Teixeira^a^, Cristina M. Cordas^b^, Marta P. Fonseca^a^, Norma E. C. Duke^c†^ P. Raj Pokkuluri^c†*^, Carlos A. Salgueiro^a*^

^a^ UCIBIO-Requimte, Departamento de Química, Faculdade de Ciências e Tecnologia, Universidade NOVA de Lisboa, Campus Caparica, 2829-516 Caparica, Portugal

^b^ LAQV, REQUIMTE, Departamento de Química, Faculdade de Ciências e Tecnologia, Universidade NOVA de Lisboa, Quinta da Torre, 2829-516 Caparica, Portugal

^c^ Biosciences Division, Argonne National Laboratory, Lemont IL, 60439, USA

*Correspondence: Carlos A. Salgueiro: [csalgueiro@fct.unl.pt](mailto:csalgueiro@fct.unl.pt); P. Raj Pokkuluri: [ppokkuluri@gmail.com](mailto:ppokkuluri@gmail.com)

†Present address:

Norma Duke, SER-CAT and the Department of Biochemistry and Molecular Biology, University of Georgia, Athens, GA 30602, USA.

P. Raj Pokkuluri, X-ray Science Division, Argonne National Laboratory, Lemont, IL 60439 USA.

The submitted manuscript has been created by UChicago Argonne, LLC, Operator of Argonne National Laboratory (“Argonne”). Argonne, a U.S. Department of Energy Office of Science laboratory, is operated under Contract No. DE-AC02-06CH11357. The U.S. Government retains for itself, and others acting on its behalf, a paid-up nonexclusive, irrevocable worldwide license in said article to reproduce, prepare derivative works, distribute copies to the public, and perform publicly and display publicly, by or on behalf of the Government.

**Supplementary Table S1.** Absorption peaks in the visible region of the electronic spectra of OmcF mutants (OmcFH47I and OmcFH47F) and wild-type OmcF.

| Protein | Peaks in the electronic absorption spectra | | | |
| --- | --- | --- | --- | --- |
|  | Soret | β | α | Other |
| OmcFH47I |  |  |  |  |
| Oxidized | 411 |  |  | 528; 563 |
| Reduced | 417 | 523 | 552 |  |
|  |  |  |  |  |
| OmcFH47F |  |  |  |  |
| Oxidized | 411 |  |  | 528; 563 |
| Reduced | 417 | 523 | 553 |  |
|  |  |  |  |  |
| OmcF |  |  |  |  |
| Oxidized | 411 |  |  | 528; 562 |
| Reduced | 417 | 522 | 552 |  |

**Supplementary Table S2.** Proton chemical shifts of the heme substituents of OmcF mutants in the reduced state (25 °C and pH 7). The chemical shifts of OmcF were previously determined (Dantas et al. 2015) and are listed for comparison.

| **Heme**  **substituent** | **Chemical shift (ppm)** | | |
| --- | --- | --- | --- |
|  | OmcF | OmcFH47I | OmcFH47F |
| 5H | 10.10 | 10.09 | 10.09 |
| 10H | 9.42 | 9.49 | 9.49 |
| 15H | 9.59 | 9.58 | 9.60 |
| 20H | 9.33 | 9.32 | 9.33 |
| 2^1^CH_3_ | 3.64 | 3.63 | 3.65 |
| 7^1^CH_3_ | 4.08 | 4.06 | 4.09 |
| 12^1^CH_3_ | 3.20 | 3.51 | 3.61 |
| 18^1^CH_3_ | 3.03 | 3.00 | 3.03 |
| 3^1^H | 5.48 | 5.49 | 5.50 |
| 8^1^H | 6.20 | 6.27 | 6.25 |
| 3^2^CH_3_ | 1.14 | 1.15 | 1.18 |
| 8^2^CH_3_ | 2.51 | 2.49 | 2.52 |
| 13^1^CH_2_ | 4.15 | 4.34 | 4.21 |
|  | 4.45 | 4.30 | 4.39 |
| 13^2^CH_2_ | 2.88 | 2.87 | 2.91 |
|  | 3.24 | 3.13 | 3.15 |
| 17^1^CH_2_ | 2.69 | 2.70 | 2.73 |
|  | 4.11 | 4.09 | 4.11 |
| 17^1^CH_2_ | 2.02 | 2.00 | 1.97 |
|  | 3.12 | 2.89 | 2.93 |

**Supplementary Table S3.** Proton chemical shifts of the heme substituents of OmcF in the reduced state at pH 6.1 and 9.4 (25 °C).

| **Heme**  **substituent** |  | **Chemical shift (ppm)** | |
| --- | --- | --- | --- |
|  |  | pH | |
|  |  | 6.1 | 9.4 |
| 5H |  | 10.11 | 10.12 |
| 10H |  | 9.42 | 9.46 |
| 15H |  | 9.60 | 9.60 |
| 20H |  | 9.35 | 9.35 |
| 2^1^CH_3_ |  | 3.65 | 3.65 |
| 7^1^CH_3_ |  | 4.08 | 4.09 |
| 12^1^CH_3_ |  | 3.14 | 3.35 |
| 18^1^CH_3_ |  | 3.04 | 3.04 |
| 3^1^H |  | 5.49 | 5.50 |
| 8^1^H |  | 6.19 | 6.23 |
| 3^2^CH_3_ |  | 1.15 | 1.17 |
| 8^2^CH_3_ |  | 2.50 | 2.51 |
| 13^1^CH_2_ |  | 4.15 | 4.19 |
|  |  | 4.48 | 4.37 |
| 13^2^CH_2_ |  | 2.90 | 2.88 |
|  |  | 3.31 | 3.11 |
| 17^1^CH_2_ |  | 2.64 | 2.86 |
|  |  | 4.17 | 3.92 |
| 17^1^CH_2_ |  | 1.97 | 1.84 |
|  |  | 3.08 | 3.26 |

**Supplementary Figure S1.** Cyclic voltammograms of OmcF and the corresponding control at a larger potential window, at 20 mV/s scan rate, in 32 mM sodium phosphate (pH 7) with 100 mM final ionic strength, shown as example. It is possible to observe an anodic process also present in the control (see black arrow) that was attributed to oxides on the working electrode surface at the more positive potentials, and one other process (cathodic) attributed to residual oxygen retained in the membrane (at lower potentials). In order to avoid these misleading processes, we have restricted the potential window range to be presented in the main manuscript text to the one where there is clearly only the redox pair that was assigned to the heme centre.


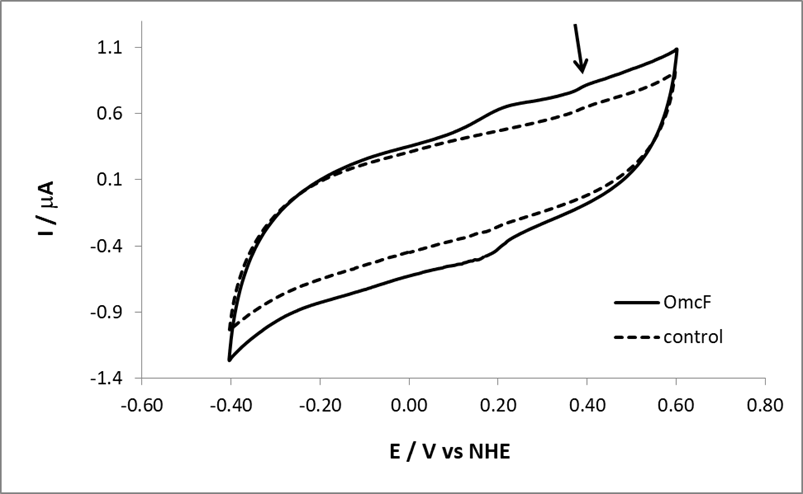


**Supplementary Figure S2.** The C_α_ atoms of the OmcFH47I mutant (orange) [this work] overlaid on the native OmcF (gray) [PDB code 3CU4 (Pokkuluri et al., 2019)]. The two structures were overlapped using the C_α_ atoms of residues 26 – 104. The figure was generated with PyMOL (The PyMOL Molecular Graphics System, Version 1.3 Schrödinger, LLC).


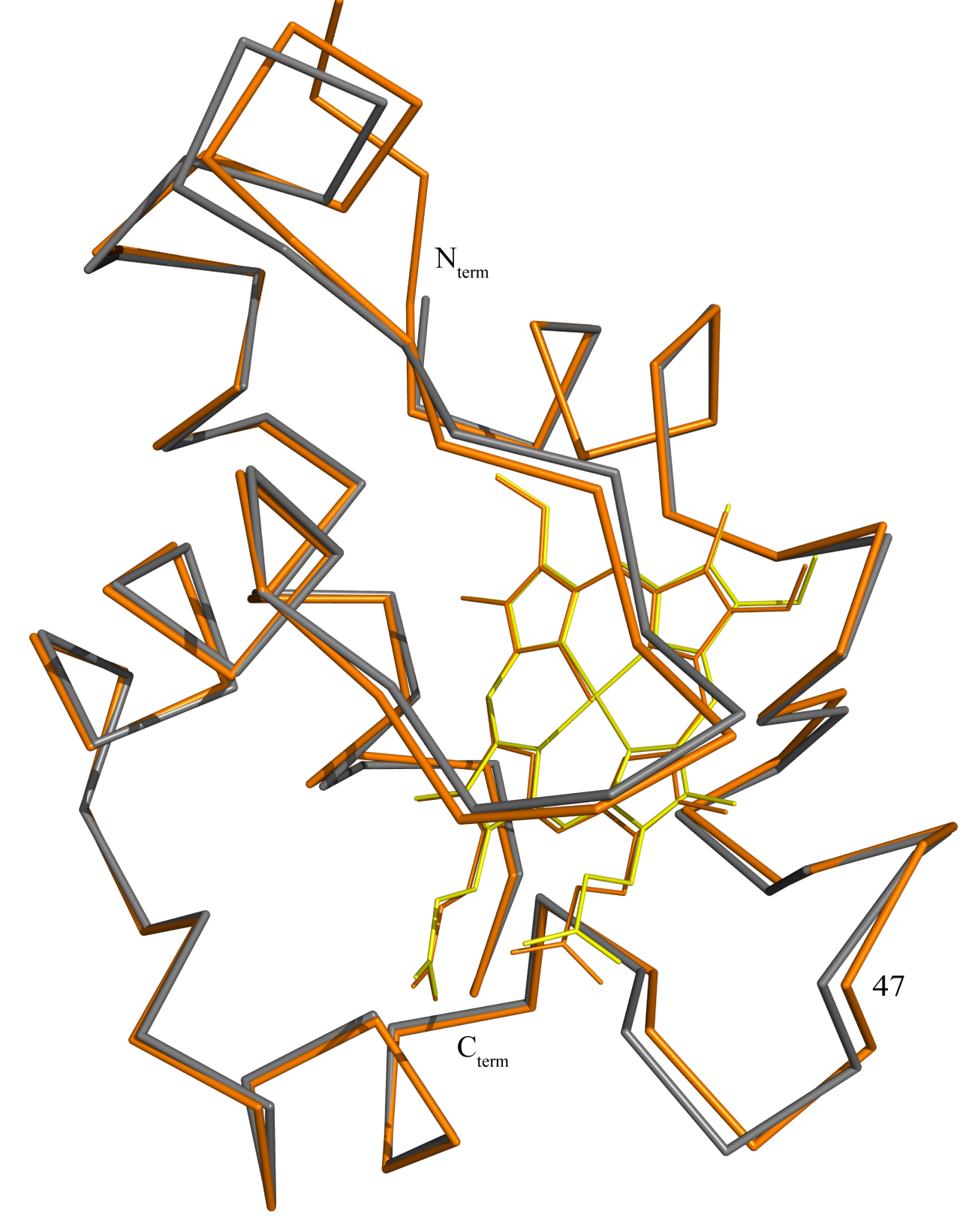

Supplement: Supplementary file 1 [file Data_Sheet_1.docx]
